# Recovery from Acute Kidney Injury and Long-Term Prognosis following Acute Myocardial Infarction

**DOI:** 10.3390/biomedicines12071490

**Published:** 2024-07-05

**Authors:** Keren Skalsky, Arthur Shiyovich, Alon Shechter, Harel Gilutz, Ygal Plakht

**Affiliations:** 1 Department of Cardiology, Rabin Medical Center, Petach Tikva 4941492, Israel; kskalsky@gmail.com (K.S.); arthur.shiyovich@gmail.com (A.S.); alonshechter@gmail.com (A.S.); 2 Faculty of Medicine, Tel Aviv University, Tel Aviv 6997801, Israel; 3 Division of Cardiovascular Medicine, Department of Medicine, Brigham and Women’s Hospital, Harvard Medical School, Boston, MA 02115, USA; 4 Faculty of Health Sciences, Ben-Gurion University of the Negev, Beer Sheva 8410501, Israel; gilutz@bgu.ac.il; 5 Department of Emergency Medicine, Soroka University Medical Center, Beer Sheva 8457108, Israel

**Keywords:** acute kidney injury, myocardial infarction, survival

## Abstract

We investigated the recovery pattern from acute kidney injury (AKI) following acute myocardial infarction (AMI) and its association with long-term mortality. The retrospective study included AMI patients (2002–2027), who developed AKI during hospitalization. Creatinine (Cr) measurements were collected and categorized into 24 h timeframes up to 7 days from AKI diagnosis. The following groups of recovery patterns were defined: rapid (24–48 h)/no rapid and early (72–144 h)/no early recovery. Specific cut-off points for recovery at each AKI stage and timeframe were determined through receiver operating characteristic (ROC) curves. The probability of long-term (up to 10 years) mortality as a post-AKI recovery was investigated using a survival approach. Out of 17,610 AMI patients, 1069 developed AKI. For stage 1 AKI, patients with a Cr ratio <1.5 at 24 h and/or <1.45 at 48 h were defined as ‘rapid recovery’; for stages 2–3 AKI, a Cr ratio <2.5 at 96 h was defined as ‘early recovery’. Mortality risk in stage 1 AKI was higher among the non-rapidly recovered: AdjHR = 1.407; 95% CI: 1.086–1.824; *p* = 0.010. Among stages 2–3 AKI patients, the risk for long-term mortality was higher among patients who did not recover in the early period: AdjHR = 1.742; 95% CI: 1.085–2.797; *p* = 0.022. The absence of rapid recovery in stage 1 AKI and lack of early recovery in stages 2–3 AKI are associated with higher long-term mortality.

## 1. Introduction

Acute kidney injury (AKI) following acute myocardial infarction (AMI), also known as cardiorenal syndrome type 1 [1], is common, occurring in approximately 14.8% to 36.6% of cases [2,3]. The underlying mechanisms contributing to AKI post-AMI are multifactorial and often include a combination of hypotension, inflammation, oxidative stress, and direct tubular injury. Previous studies have shown post-AMI AKI to be independently associated with increased morbidity and mortality, such that patients experiencing complications are at increased risk of developing chronic kidney disease (CKD), end-stage kidney disease (ESKD), and adverse cardiovascular events [3,4,5,6,7,8,9].

Recovery from post-AMI AKI is variable and influenced by the severity of renal injury, the promptness of treatment, and the patient’s background. While in many cases, there is an initial improvement in renal function within the first week, and a full recovery by several weeks to months, a subset of patients may not regain their baseline renal function, leading to CKD and its associated long-term sequelae [10,11].

The aim of the current study is to characterize AKI following AMI based on the time to recovery and to assess its association with long-term prognosis.

## 2. Materials and Methods

### 2.1. Study Population

Our research relies on data from the Soroka Acute Myocardial Infarction (SAMI) registry, encompassing consecutive hospitalizations for AMI occurring at Soroka University Medical Center (SUMC), Israel, from 1 January 2002 to 31 October 2017 [12,13]. We included adult (i.e., ≥18-year-old) Israeli citizens who were hospitalized due to AMI at SUMC, developed AKI during their hospitalization, and were discharged alive. Additional inclusion criteria were estimated glomerular filtration rate (eGFR, ml/min/1.73 m^2^) >60 at admission and available data regarding at least two serum creatinine (Cr) tests during hospitalization: upon admission and at days 3 to 7. Patients with a diagnosis of hemodialysis/peritoneal dialysis at baseline and patients with a hospitalization period of less than three days were excluded from the analysis.

This project conformed to the Declaration of Helsinki and was approved by SUMC’s Institutional Review Board, which waived the need for informed consent in view of the investigation’s retrospective nature.

### 2.2. Follow-Up and Outcome

The study period spanned the timeframe between hospital discharge and either death or 30 December 2021. The study outcome was all-cause mortality up to 10 years after hospital discharge. Death events and dates were obtained from the Israeli Ministry of the Interior Population Registry.

### 2.3. Data Collection and Definitions

Patients’ clinical data were retrieved using electronic medical records. Baseline comorbidities were identified by International Classification of Diseases, Ninth Revision, Clinical Modification (ICD-9-CM) codes, as documented in real time by the treating medical team. Laboratory results and echocardiographic and angiographic findings were documented.

Patients hospitalized with AMI were allocated according to ICD-9-CM code 410. AMI diagnosis was based on ischemic signs and/or symptoms coupled with abrupt rise and fall in cardiac biomarkers levels consistent with acute myocardial injury, as dictated by the Universal Definition of Myocardial Infarction at the time (if applicable). Obstructive coronary artery disease (CAD) required the presence of a ≥70% vessel stenosis as assessed by angiography.

### 2.4. Study Strata and Groups

The first Cr measurement reported within the initial 24 h of admission was considered as the baseline Cr level for analysis. AKI was defined as an absolute increase in Cr ≥ 0.3 mg/dL (≥26.5 μmol/L) within 48 h or a relative increase in Cr to ≥1.5 times within 7 days, compared with the baseline level. The study strata, in accordance with AKI stage, was defined as 1 to 3 according to the following criteria: stage 1—an absolute increase in Cr ≥ 0.3 mg/dL or a relative increase of 1.5–1.9 from baseline; stage 2—a relative increase of 2–2.9 times baseline; and stage 3—an absolute increase to ≥4.0 mg/dL (≥353.6 μmol/L), a relative increase to 3.0 times baseline or initiation of renal replacement therapy [14,15,16].

All Cr measurements taken during hospitalization were retrieved, and categorized into 24 h timeframes, from the diagnosis of AKI and up to 7 days. Every time point encompassed all Cr tests conducted within a 12 h window both preceding and following that specific point. For instance, the 24 h interval comprised tests administered between 12 and 36 h. The recovery measure was calculated for each patient by dividing the Cr at a given time point by the baseline Cr level (Cr ratio). The recovery timeframes were defined as ‘rapid’, characterized as occurring within 24–48 h, and ‘early’, designated for the 72–144 h timeframe. Those patterns were based on previous publications referring to AKI recovery within 7 days as ‘early’ [17].

### 2.5. Statistical Analysis

All statistical analyses were performed separately for each study’s strata. The variables were reported as frequencies and percentages, medians, and interquartile ranges (IQRs), or means and standard deviations/standard errors (SD/SE), as appropriate. A comparison of the baseline characteristics between the patients who died and survivors was performed using Pearson’s Chi-Squared test for categorical variables and Student’s *t* test for count data. Particularly, using Student’s *t* tests, we compared the mean Cr rates for each timeframe.

The discriminative power of the Cr ratio was calculated using logistic regressions and receiver operating characteristic (ROC) curves. Based on these analyses, a cut-off point was calculated for each timeframe in which the Cr ratio was found to be significantly different among those who survived compared to those who died. In accordance with the cut-off point values and the timeframes, the following study groups were defined: rapid recovery/no rapid recovery and early recovery/no early recovery.

The probability of mortality as a post-AKI recovery was graphically displayed according to the survival approach (Kaplan–Meier method), and compared using the Log-rank test. Additionally, univariable and multivariable Cox regression analyses were utilized. The results of the models were expressed as Hazard Ratios/Adjusted Hazard Ratios (HR/AdjHR) and 95% Confidence Intervals (CI). The multivariable regressions incorporated the parameters of recovery and the baseline variables demonstrating a *p*-value of <0.1 on a preliminary univariable model. Statistical significance required a two-sided *p*-value of <0.05. All analyses were performed using SPSS, version 29 (IBM Corporation, Armonk, NY, USA).

## 3. Results

### 3.1. Study Population and Strata

During the years 2002 and 2017, 17,610 patients were admitted to SUMC with AMI. Of them, 10,555 were eligible for analysis according to the inclusion and exclusion criteria, and 1140 (10.8%) developed AKI during their hospitalization. Seventy-one (71) of them had no documented follow-up Cr tests, and were excluded from the analysis. Thus, 1069 patients were included in the final cohort (Supplementary Figure S1). Among them, 835 (78.1%) had stage 1 AKI, and 185 (17.3%) and 49 (4.6%) had stages 2 and 3 AKI, respectively. Due to the relatively low number of patients experiencing stage 2 and 3 AKI, the two groups were combined for analysis (n = 234).

The total mortality rate was (402/835 = 51.9%) among the patients with stage 1 AKI, whereas in stages 2–3 AKI, the incidence of mortality was higher (147/234 = 62.8%), *p* < 0.001.

### 3.2. Baseline Characteristics in Accordance with Status of Death

Baseline characteristics according to the AKI stage and the survival status are summarized in Table 1. Older patients with AKI at any stage were more likely to die. Additionally, heart diseases including cardiomegaly, congestive heart failure, and pulmonary heart disease, and conventional cardiovascular risk factors, including diabetes mellites, dyslipidemia, hypertension, smoking and peripheral vascular disease were found to be associated with increased mortality.

### 3.3. Baseline Characteristics in Accordance with Status of Death

**Figure 1:**
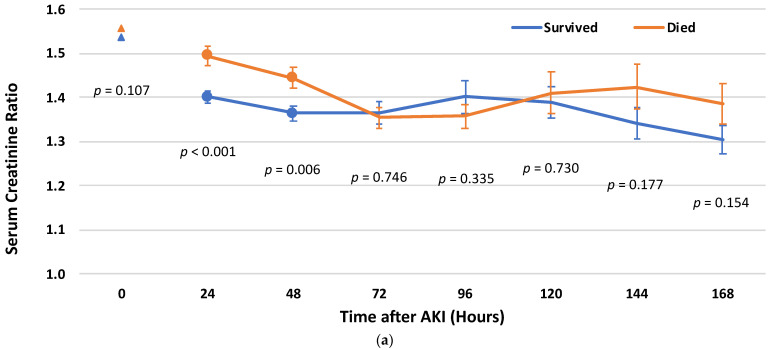

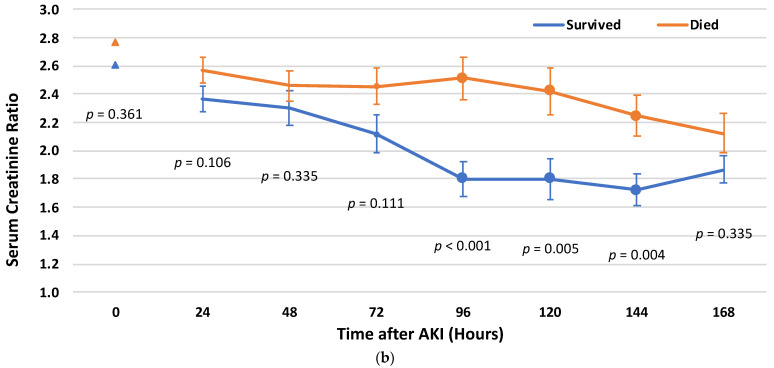
Serum Creatinine ratio (mean and standard error) by timeframe (hours after Acute kidney injury [AKI]) among the patients who died and among survivors: (**a**) among the patients with AKI stage 1; (**b**) among the patients with AKI stages 2 and 3.

Rapid recovery from stage 1 AKI was associated with a higher rate of long-term survival. The mean ± SD Cr ratio at 24 h among those who died was 1.49 ± 0.49, compared to 1.40 ± 0.31 among patients who survived (*p* < 0.001); and at 48 h, 1.44 ± 0.44 compared to 1.36 ± 0.37 (*p* = 0.006). No significant differences in the Cr ratio values between the patients who died and the survivors were found after 48 h (Figure 1a).

At stages 2–3 AKI, the differences in Cr ratio values became statistically significant in the early period after the AKI event. The mean ± SD Cr ratios among the patients who died, as compared with the survivors at the time periods of 96, 120, and 144 h, were 2.51 ± 1.62 vs. 1.80 ± 1.0 (*p* < 0.001), 2.42 ± 1.71 vs. 1.80 ± 0.95 (*p* = 0.005), and 2.25 ± 1.43 vs. 1.72 ± 0.69 (*p* = 0.004), respectively (Figure 1b).

### 3.4. Cr Rate Cut-Off Points for Post-AKI Recovery

The discrimination of the Cr ratio between the non-survivals and the survivals was based on ROC curves. A separate ROC curve was built for each timeframe in which there was a statistically significant difference in the Cr ratio between the patients who died and the survivors. For stage 1 AKI, a higher risk for death was associated with Cr ratio cut-off points of 1.50 for 24 h recovery (Odds Ratio [OR] = 1.384; 95% CI: 0.997–1.922; *p* = 0.052; sensitivity 42.9% and specificity 64.8%) and 1.45 for 48 h recovery (OR = 1.574; 95% CI: 1.090–2.273; *p* = 0.016, sensitivity 43.6% and specificity 67.0%).

For the patients with AKI stages 2–3, a Cr ratio cut-off point of 2.5 for 96 h recovery was associated with a higher risk of death (OR = 6.034; 95% CI: 1.998–18.219; *p* = 0.001, sensitivity 38.8% and specificity 90.5%).

### 3.5. Survival Analysis

Median follow-up was 2470 (IQR 1278–6352) days for stage 1 AKI and 1925 (514–3435) for stages 2–3 AKI. Cumulative mortalities were 0.523 and 0.676 for AKI stages 1 and 2–3, respectively.

Survival analysis compared the probability of dying during the follow-up period in the different groups of patients. Based on the previously defined cut-off points and the timeframes for AKI recovery, three groups of patients for each study strata were defined. Particularly, for stage 1 AKI—patients with a Cr ratio <1.5 at 24 h and/or <1.45 at 48 h were defined as ‘rapid recovery’ (619 patients). Those with a Cr ratio ≥1.5 within 24 h and a Cr ratio ≥1.45 within 48 h were defined as ‘not rapidly recovered’ (141 patients). The third group included patients with missing values of Cr within 24–48 h (75 patients). The baseline characteristics of the patients by the study groups are presented in Supplementary Table S1a. Females, along with individuals having diabetes mellitus and obesity, had a lower probability of achieving rapid recovery. The treatment approach varied between the two groups, with a higher incidence of percutaneous coronary intervention (PCI) among those who recovered rapidly, while the rate of surgical treatment (coronary artery bypass graft—CABG) was lower in this group.

For stages 2–3 AKI patients, a Cr ratio <2.5 at 96 h was defined as ‘early recovery’ (98 patients), and a Cr ratio ≥2.5 at this time was defined as ‘not early recovered’ (32 patients). The third group included those with missing values at this time (102 patients) (Supplementary Table S1b). Patients with diabetes mellitus and anemia demonstrated a reduced likelihood of experiencing early recovery, while the treatment strategy was similar in both groups.

**Figure 2:**
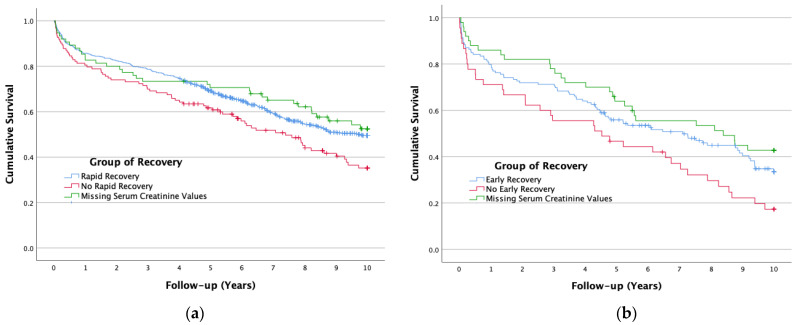
Cumulative long-term survival of the patients after Acute kidney injury (AKI) by the group of recovery: (**a**) among the patients with AKI stage 1; (**b**) among the patients with AKI stages 2 and 3.

Among the patients with stage 1 AKI, cumulative mortality was significantly higher in the group with no rapid recovery compared to the group with rapid recovery (0.630 vs. 0.505; *p* = 0.024). Cumulative mortality for the patients with missing values of Cr within this timeframe was 0.475, with no statistically significant differences from the group of rapid recovery (*p* = 0.580) (Figure 2a).

At stages 2–3 of AKI, cumulative mortality was higher among patients with no early recovery compared to those with early recovery (0.853 vs. 0.619; *p* = 0.024). Cumulative mortality for the patients with missing values of Cr within this timeframe was 0.655, with no statistically significant difference from the group of early recovery (*p* = 0.580) (Figure 2b).

### 3.6. Post-AKI Recovery and the Risk for Mortality

Among stage 1 AKI patients, the risk for long-term mortality in the no rapid recovery group was higher than the risk of patients with rapid recovery: HR = 1.331; 95% CI: 1.037–1.707; *p* = 0.025. The risk of patients with missing values of Cr within this timeframe was not different from the rapid recovery group: HR = 0.902; 95% CI: 0.632–1.287; *p* = 0.669.

Among stages 2–3 AKI patients, the risk for long-term mortality was higher among patients with no early recovery, compared to those with early recovery: HR = 1.755; 95% CI: 1.117–2.757; *p* = 0.015. The risk of patients with missing values of Cr within this timeframe was not different from the early recovery group: HR = 1.007; 95% CI: 0.701–1.447; *p* = 0.970.

After adjustment for the investigated confounders, the risk for long-term all-cause mortality has remained statistically significantly higher for those who did not recover rapidly or early compared with those who did. Among stage 1 AKI patients, no rapid recovery vs. rapid recovery, AdjHR was 1.407; 95% CI: (1.086–1.824); *p* = 0.010 (Table 2a). Accordantly, among stages 2–3 AKI, no early recovery vs. early recovery, the AdjHR was 1.742; 95% CI: 1.085–2.797; *p* = 0.022 (Table 2b).

## 4. Discussion

The present study investigated the phenomenon of AKI following AMI by the time to recovery and its association with all-cause long-term mortality. We have addressed the different AKI stages and recovery, according to a unique calculation, based on the baseline Cr of each patient. We found that patients with stages 2–3 AKI had higher mortality rates compared to patients with AKI stage 1. Next, we have characterized kidney recovery in association with long-term mortality after AMI associated with AKI. We found that patients with stage 1 AKI who experienced rapid recovery from the event (as compared with no rapid recovery), and patients with stages 2–3 AKI who experienced early recovery (as compared to no early recovery), had higher survival. We have found Cr ratio cut-off points for each AKI stage and timeframe: for stage 1 AKI—1.5 at 24 h and 1.45 at 48 h; for stages 2–3 AKI—2.5 at 96 h. Increased values of the Cr ratio above the cut-off points were found to be associated with a higher risk of death. There were two differences between stage 1 AKI and stages 2 and 3 AKI: first, the prognostic significance of kidney recovery in AKI stages 2–3 was only found after 96 h from the injury (early recovery), and second, the Cr ratios above these cut-off points were found to be associated with increased mortality. As outlined in the methods, each time point included all Cr tests performed within a 12 h period before and after that particular point.

The mechanism of the cardiorenal syndrome type 1 is complex. The syndrome is initiated through the combination of renal congestion, diminished renal perfusion and activation of the renin–angiotensin–aldosterone system, followed by inflammatory cell infiltration and direct tubular damage [1,18]. Several risk factors have been identified in the literature that increase the likelihood of AKI development, including advanced age, pre-existing kidney disease, diabetes mellitus, anemia, heart failure, and the use of nephrotoxic medications and high doses of contrast agents [19].

The mechanism of the renal repair process is based on hemodynamic stabilization and improved heart function accompanied by the renal regenerative process and repopulation of the surviving tubular cells, along with paracrine growth factor secretion [17,20]. In specific situations, renal restoration can become counterproductive, resulting in inflammation, fibrosis, and vascular rarefaction that all contribute to ongoing cellular and tissue dysfunction, ultimately culminating in CKD.

A rapid recovery from stage 1 AKI associated with a better prognosis is expected, as the kidney injury is transient and does not cause damage for more than a short period of time. The prognostic significance of the early recovery from stages 2–3 AKI can be explained in a different way—the initial injury is significant and somewhat extreme and the healing process lasts more than a couple of days. The success of this healing is revealed after 96 h, and this is the timeframe in which the Cr rate becomes significant. The higher Cr ratio cut-off point in stages 2–3 AKI is logical, as it represents the initial harm to the kidney function, which is more prominent than in stage 1 AKI. In both cases, kidney recovery implies the presence of premorbid renal reserve, which enables the adaptive repair process.

The terms renal recovery and time pattern are not clearly defined in the literature. Some studies define kidney recovery as a return to normal kidney function, while others address it as Cr decline and improvement in the kidney disease stage [21]. As for the time pattern—it varies, as early recovery can be defined as one that occurs within 72 h [17], 3–7 days [22], up to 10 days [23], or when there is ‘in-hospital recovery’ [11].

The KDIGO guideline characterizes AKI as an abrupt decline in kidney function within a span of 7 days or less, whereas CKD is defined by the enduring presence of kidney disease for a period exceeding 90 days, and renal injury present between days 7 to 90 is referred as acute kidney disease [15].

Our definition of the recovery as rapid (within 2 days) and early (3–6 days) is a combination of the above definitions. As for the recovery itself, we have used the serial Cr measurements and calculation of the Cr ratio, according to the Acute Disease Quality Initiative (ADQI) consensus report on AKI and renal recovery [21]. The calculation was based on the equation, in which the serum Cr following the AKI event is divided by the baseline Cr. The use of this method has the advantage of addressing each patient’s baseline Cr and their personal improvement. It can be simply calculated and used in the clinical evaluation of patients with AKI following AMI.

Early recognition and management of AKI are crucial for improving outcomes post-AMI. Timely intervention involves adequate hemodynamic support, discontinuation of nephrotoxic medications and optimizing volume status to enhance renal perfusion.

Moreover, the development of new therapeutic agents that provide both cardiovascular and renal benefits can be considered, as those can potentially act as disease modifiers, meaning they not only manage the symptoms, but also address the underlying causes of the illness. The medications that have been explored in this context include renin–angiotensin–aldosterone System (RAAS) inhibitors, sodium–glucose cotransporter 2 (SGLT2) inhibitors, mineralocorticoid receptor antagonists and GLP-1 receptor antagonists. Our study results enable identifying populations with a greater potential for kidney recovery and may guide physicians in their clinical decisions.

### Limitations

The study exhibits several limitations. The primary constraint lies in its retrospective design and the derivation of the cohort from a single center. However, this cohort stands out as one of the largest reported to date, and its findings, particularly regarding AKI, align with prior publications, thereby bolstering the study’s validity. Secondly, the groups of patients with AKI stages 2 and 3 were relatively small, necessitating their combination for analysis. Additionally, a significant number of patients in these groups had missing values, potentially impacting the interpretation of results. Thirdly, our cohort lacks information on medical therapies administered during hospitalization and post-discharge, the number of radiocontrast agents given to each patient, and specific details about causes of death and other cardiovascular and renal outcomes. However, adjustments for confounding factors have been made, and the risk for the primary outcome, long-term all-cause mortality, remains statistically significant.

## 5. Conclusions

The findings from our study indicate an association between the AKI stage following AMI and the recovery patern from AKI with the risk of long-term all-cause mortality. Long-term follow-up reveal that patients experiencing stage 1 AKI without recovery within the initial 48 h and those with stages 2–3 AKI without recovery within 96 h are at a higher risk of mortality. We established specific cut-off points to address recovery at each AKI stage and timeframe. Pending further research, early intervention for AKI during the initial phase among AMI patients may offer potential benefits.

## Figures and Tables

**Table 1:** Baseline characteristics and survival status: (**a**) among the patients with acute kidney injury (AKI) stage 1; (**b**) among the patients with AKI stages 2 and 3.

(a)				
Group	Survived	Died	Total	*p*
n	433	402	835	
**Demographics**				
Age, Years, Mean (SD)	62.05 (10.51)	71.95 (10.63)	66.82 (11.66)	<0.001
<65	273 (63)	111 (27.6)	384 (46.0)	<0.001
65–75	108 (24.9)	129 (32.1)	237 (28.4)	
≥75	52 (12.0)	162 (40.3)	214 (25.6)	
Sex, Male	367 (84.8)	293 (72.9)	660 (79.0)	<0.001
Ethnicity, Arab/Other	74 (17.1)	72 (17.9)	146 (17.5)	0.755
**Cardiac diseases**				
Cardiomegaly	46 (10.6)	65 (16.2)	111 (13.3)	0.018
Supraventricular arrhythmias	44 (10.2)	113 (28.1)	157 (18.8)	<0.001
Congestive heart failure	77 (17.8)	149 (37.1)	226 (27.1)	<0.001
Pulmonary heart disease	21 (4.8)	53 (13.2)	74 (8.9)	<0.001
Chronic ischemic heart disease	410 (94.7)	345 (85.8)	755 (90.4)	<0.001
Previous myocardial infarction	65 (15.0)	120 (29.9)	185 (22.2)	<0.001
Previous percutaneous coronary intervention	85 (19.6)	101 (25.1)	186 (22.3)	0.057
Previous coronary artery bypass graft	22 (5.1)	55 (13.7)	77 (9.2)	<0.001
Atrioventricular block	19 (4.4)	17 (4.2)	36 (4.3)	0.910
**Cardiovascular risk factors**				
Diabetes mellitus	178 (41.1)	230 (57.2)	408 (48.9)	<0.001
Dyslipidemia	376 (86.8)	326 (81.1)	702 (84.1)	0.023
Hypertension	267 (61.7)	297 (73.9)	564 (67.5)	<0.001
Obesity	112 (25.9)	103 (25.6)	215 (25.7)	0.936
Smoking	207 (47.8)	151 (37.6)	358 (42.9)	0.003
Peripheral vascular disease	39 (9.0)	101 (25.1)	140 (16.8)	<0.001
Family history of ischemic heart disease	64 (14.8)	13 (3.2)	77 (9.2)	<0.001
**Other disorders**				
Chronic obstructive pulmonary disease	17 (3.9)	70 (17.4)	87 (10.4)	<0.001
Neurological disorders	52 (12.0)	93 (23.1)	145 (17.4)	<0.001
Malignancy	9 (2.1)	33 (8.2)	42 (5.0)	<0.001
Anemia	265 (61.2)	269 (66.9)	534 (64.0)	0.086
Gastrointestinal bleeding	14 (3.2)	24 (6.0)	38 (4.6)	0.058
Schizophrenia/Psychosis	8 (1.8)	15 (3.7)	23 (2.8)	0.097
Alcohol/Drug addiction	7 (1.6)	16 (4.0)	23 (2.8)	0.037
History of malignancy	18 (4.2)	25 (6.2)	43 (5.1)	0.178
**Administrative characteristics of the hospitalization**				
Admitted/transposed to ICCU	345 (79.7)	244 (60.7)	589 (70.5)	<0.001
Length of hospital stay, days, Mean (SD)	16.46 (10.04)	18.55 (14.87)	17.47 (12.64)	0.017
≥7	375 (86.6)	335 (83.3)	710 (85.0)	0.185
**Clinical characteristics of the hospitalization**				
Type of AMI, STEMI	234 (54.0)	154 (38.3)	388 (46.5)	<0.001
**Results of echocardiography**				
Echocardiography performance	365 (84.3)	309 (76.9)	674 (80.7)	0.007
Severe left ventricular dysfunction	53 (14.5)	76 (24.6)	129 (19.1)	0.001
Left ventricular hypertrophy	13 (3.6)	22 (7.1)	35 (5.2)	0.038
Mitral regurgitation	17 (4.7)	37 (12.0)	54 (8.0)	<0.001
Tricuspid regurgitation	7 (1.9)	22 (7.1)	29 (4.3)	0.001
Pulmonary hypertension	17 (4.7)	52 (16.8)	69 (10.2)	<0.001
**Results of angiography**				
Angiography performance	357 (82.4)	251 (62.4)	608 (72.8)	<0.001
Measure of coronary artery disease				
No/non-significant	4 (1.1)	5 (2.0)	9 (1.5)	0.175
One vessel	48 (13.4)	24 (9.6)	72 (11.8)	
Two vessels	73 (20.4)	43 (17.1)	116 (19.1)	
Three vessels/Left main artery	232 (65.0)	179 (71.3)	411 (67.6)	
**Type of treatment**				
Noninvasive	11 (2.5)	110 (27.4)	121 (14.5)	<0.001
Percutaneous coronary intervention	142 (32.8)	140 (34.8)	282 (33.8)	
Coronary artery bypass graft	280 (64.7)	152 (37.8)	432 (51.7)	
**Kidney function**				
eGFR (first), Mean (SD)	84.30 (20.00)	80.80 (18.09)	82.62 (19.17)	0.008
Creatinine at AKI, Mean (SD)	1.20 (0.25)	1.27 (0.25)	1.23 (0.25)	<0.001
**(b)**				
**Group**	**Survived**	**Died**	**Total**	***p***
n	87	147	234	
**Demographics**				
Age, Years, Mean (SD)	62.33 (11.70)	71.39 (11.39)	68.02 (12.29)	<0.001
<65	48 (55.2)	38 (25.9)	86 (36.8)	<0.001
65–75	28 (32.2)	49 (33.3)	77 (32.9)	
≥75	11 (12.6)	60 (40.8)	71 (30.3)	
Sex, Male	59 (67.8)	77 (52.4)	136 (58.1)	0.021
Ethnicity, Arab/Other	12 (13.8)	22 (15.0)	34 (14.5)	0.806
**Cardiac diseases**				
Cardiomegaly	8 (9.2)	29 (19.7)	37 (15.8)	0.033
Supraventricular arrhythmias	16 (18.4)	47 (32.0)	63 (26.9)	0.024
Congestive heart failure	17 (19.5)	60 (40.8)	77 (32.9)	0.001
Pulmonary heart disease	6 (6.9)	28 (19.0)	34 (14.5)	0.011
Chronic ischemic heart disease	81 (93.1)	111 (75.5)	192 (82.1)	0.001
Previous myocardial infarction	15 (17.2)	44 (29.9)	59 (25.2)	0.031
Previous percutaneous coronary intervention	16 (18.4)	27 (18.4)	43 (18.4)	0.996
Previous coronary artery bypass graft	3 (3.4)	20 (13.6)	23 (9.8)	0.012
Atrioventricular block	4 (4.6)	8 (5.4)	12 (5.1)	0.777
**Cardiovascular risk factors**				
Diabetes mellitus	37 (42.5)	90 (61.2)	127 (54.3)	0.006
Dyslipidemia	71 (81.6)	118 (80.3)	189 (80.8)	0.802
Hypertension	51 (58.6)	108 (73.5)	159 (67.9)	0.019
Obesity	19 (21.8)	39 (26.5)	58 (24.8)	0.422
Smoking	42 (48.3)	48 (32.7)	90 (38.5)	0.018
Peripheral vascular disease	12 (13.8)	39 (26.5)	51 (21.8)	0.023
Family history of ischemic heart disease	8 (9.2)	6 (4.1)	14 (6.0)	0.111
**Other disorders**				
Chronic obstructive pulmonary disease	6 (6.9)	31 (21.1)	37 (15.8)	0.004
Neurological disorders	14 (16.1)	49 (33.3)	63 (26.9)	0.004
Malignancy	3 (3.4)	8 (5.4)	11 (4.7)	0.486
Anemia	47 (54.0)	113 (76.9)	160 (68.4)	<0.001
Gastrointestinal bleeding	4 (4.6)	9 (6.1)	13 (5.6)	0.772
Schizophrenia/Psychosis	1 (1.1)	4 (2.7)	5 (2.1)	0.422
Alcohol/Drug addiction	1 (1.1)	1 (0.7)	2 (0.9)	0.706
History of malignancy	3 (3.4)	6 (4.1)	9 (3.8)	0.808
**Administrative characteristics of the hospitalization**				
Admitted/transposed to ICCU	76 (87.4)	91 (61.9)	167 (71.4)	<0.001
Length of hospital stay, days, Mean (SD)	19.37 (32.82)	32.82 (28.18)	27.82 (24.46)	<0.001
≥7	81 (93.1)	139 (94.6)	220 (94.0)	0.650
**Clinical characteristics of the hospitalization**				
Type of AMI, STEMI	42 (48.3)	52 (35.4)	94 (40.2)	0.052
**Results of echocardiography**				
Echocardiography performance	73 (83.9)	112 (76.2)	185 (79.1)	0.161
Severe left ventricular dysfunction	13 (17.8)	35 (31.3)	48 (25.9)	0.041
Left ventricular hypertrophy	4 (5.5)	8 (7.1)	12 (6.5)	0.653
Mitral regurgitation	3 (4.1)	8 (7.1)	11 (5.9)	0.394
Tricuspid regurgitation	2 (2.7)	9 (8.0)	11 (5.9)	0.205
Pulmonary hypertension	4 (5.5)	15 (13.4)	19 (10.3)	0.083
**Results of angiography**				
Angiography performance	74 (85.1)	88 (59.9)	162 (69.2)	<0.001
Measure of coronary artery disease				
No/non-significant	2 (2.7)	4 (4.5)	6 (3.7)	0.214
One vessel	16 (21.6)	10 (11.4)	26 (16.0)	
Two vessels	16 (21.6)	17 (19.3)	33 (20.4)	
Three vessels/Left main artery	40 (54.1)	57 (64.8)	97 (59.9)	
**Type of treatment**				
Noninvasive	3 (3.4)	49 (33.3)	52 (22.2)	<0.001
Percutaneous coronary intervention	30 (34.5)	49 (33.3)	79 (33.8)	
Coronary artery bypass graft	54 (62.1)	49 (33.3)	103 (44.0)	
**Kidney function**				
eGFR (first), Mean (SD)	113.34 (109.81)	91.98 (44.88)	99.92 (76.29)	0.086
Creatinine at AKI, Mean (SD)	1.71 (1.00)	1.82 (1.15)	1.78 (1.10)	0.434

Data are presented as numbers (percentage), unless specified otherwise. AKI—Acute kidney injury, AMI—Acute myocardial infarction, eGFR—Estimated glomerular filtration rate (mL/min/1.73 m^2^), ICCU—Intensive cardiac care unit, SD—standard deviation, STEMI—ST-Elevation myocardial infarction.

**Table 2:** Relationship between kidney recovery and the risk for long-term all-cause mortality—multivariable analysis: (**a**) among the patients with acute kidney injury (AKI) stage 1; (**b**) among the patients with AKI stages 2 and 3.

(a)				
Parameter	B (SE)	AdjHR	(95% CI)	*p*
Recovery group *:				
Rapid recovery		1 (ref.)		
No rapid recovery	0.341 (0.132)	1.407	(1.086–1.824)	0.010
Missing values of serum Creatinine	−0.023 (0.184)	0.978	(0.681–1.403)	0.903
Age (years): ≥75 vs. <75	0.771 (0.113)	2.162	(1.731–2.700)	<0.001
Supraventricular arrhythmias	0.379 (0.116)	1.461	(1.163–1.836)	0.001
Congestive heart failure	0.387 (0.111)	1.473	(1.186–1.831)	<0.001
Previous myocardial infarction	0.383 (0.116)	1.467	(1.169–1.840)	<0.001
Previous coronary artery bypass graft	0.376 (0.153)	1.457	(1.079–1.968)	0.014
Diabetes mellitus	0.470 (0.108)	1.600	(1.296–1.976)	<0.001
Chronic obstructive pulmonary disease	0.938 (0.140)	2.554	(1.940–3.362)	<0.001
Malignancy	0.657 (0.190)	1.929	(1.330–2.797)	<0.001
Schizophrenia/Psychosis	0.746 (0.271)	2.109	(1.241–3.584)	0.006
Alcohol/Drug addiction	1.084 (0.262)	2.955	(1.770–4.934)	<0.001
Type of treatment:				
Type of AMI: NSTEMI vs. STEMI	0.200 (0.109)	1.221	(0.986–1.513)	0.067
Noninvasive		1 (ref.)		
Percutaneous coronary intervention	−0.947 (0.138)	0.338	(0.296–0.509)	<0.001
Coronary artery bypass graft	−1.326 (0.139)	0.265	(0.202–0.349)	<0.001
**(b)**				
**Parameter**	**B (SE)**	**AdjHR**	**(95% CI)**	***p***
Recovery group *:				
Early recovery		1 (ref.)		
No early recovery	0.555 (0.242)	1.742	(1.085–2.797)	0.022
Missing values of serum Creatinine	−0.022 (0.189)	0.978	(0.676–1.416)	0.908
Age (years): ≥75 vs. <75	0.969 (0.179)	2.636	(1.856–3.743)	<0.001
Cardiomegaly	1.137 (0.225)	3.117	(2.007–4.841)	<0.001
Diabetes mellitus	0.817 (0.190)	2.263	(1.560–3.282)	<0.001
Neurological disorders	0.472 (0.182)	1.603	(1.123–2.290)	0.009
Malignancy	0.830 (0.375)	2.294	(1.099–4.788)	0.027
Type of treatment:				
Noninvasive		1 (ref.)		
Percutaneous coronary intervention	−1.039 (0.214)	0.354	(0.233–0.538)	<0.001
Coronary artery bypass graft	−1.777 (0.226)	0.169	(0.109–0.263)	<0.001

* Recovery groups were defined as: ‘Rapid recovery’—patients with creatinine ratio <1.5 at 12–36 h and/or <1.45 at 36–60 h after AKI diagnosis; ‘No rapid recovery’—patients with creatinine ratio ≥1.5 at 12–36 h and ≥1.45 at 36–60 h after AKI diagnosis; ‘Early recovery’—patients with creatinine ratio <2.50 at 84–108 h after AKI diagnosis; ‘No early recovery’—patients with creatinine ratio ≥2.50 at 84–108 h after AKI diagnosis. AdjHR—Adjusted hazard ratio, AKI—Acute kidney injury, B—Regression coefficient, CI—Confidence interval, NSTEMI—Non-ST-elevation myocardial infarction, STEMI- ST-elevation myocardial infarction, Ref.—Reference group, SE—Standard error.

## Data Availability

The data presented in this study are available on request from the corresponding author. The data are not publicly available due to privacy or ethical restrictions.

## Supplementary Materials

The following supporting information can be downloaded at: https://www.mdpi.com/article/10.3390/biomedicines12071490/s1, Figure S1: The study flow chart; Table S1: Baseline characteristics of patients by Acute kidney injury (AKI) recovery group.
