# Supplementary material for: Recovery from Acute Kidney Injury and Long-Term Prognosis following Acute Myocardial Infarction
Source: Biomedicines. 2024 Jul 5;12(7):1490. doi: 10.3390/biomedicines12071490 (PMC11274707; doi:10.3390/biomedicines12071490)
Supplement: Supplementary file 1 [file biomedicines-12-01490-s001.zip › biomedicines-3066468-supplementary.pdf]

Supplementary Figure S1. The study flow chart.

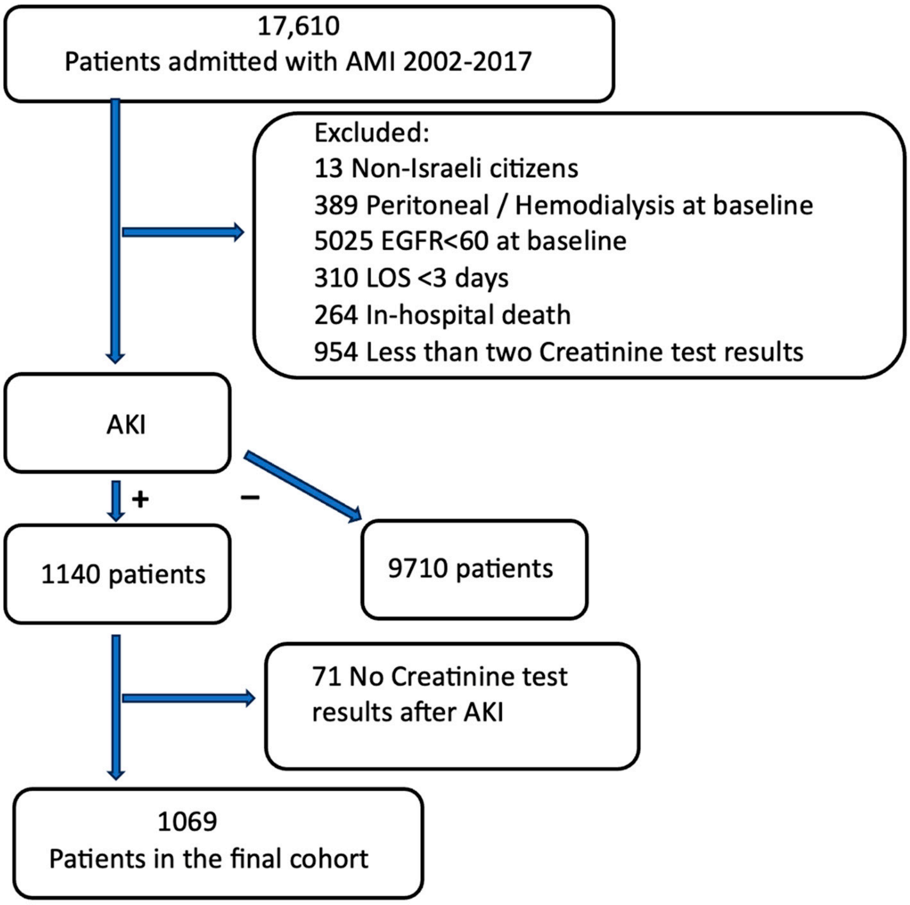

AKI – Acute kidney injury, AMI – Acute myocardial infarction, eGFR - Estimated glomerular filtration rate (ml/min/1.73m^2), LOS – length of hospital stay,

Supplementary Table S1. Baseline characteristics of the patients by Acute kidney injury (AKI) recovery group: **(a)** among the patients with AKI stage 1; **(b)** among the patients with AKI stages 2 and 3.

(a)

Recovery group*	(1) Rapid recovery	(2) No rapid recovery	(3) Missing values of serum Creatinine	Total	p (Overall)	p (1 vs. 2)
n	619	141	75	835		
Demographics						
Age, Years, Mean (SD)	66.87 (11.95)	67.33 (10.53)	65.43 (11.29)	65.82 (11.61)	0.509	0.669
<65	284 (45.9)	63 (44.7)	37 (49.3)	384 (46.0)		
65-75	171 (27.6)	42 (29.8)	24 (32.0)	237 (28.4)	0.262	0.875
≥75	164 (26.5)	36 (25.5)	14 (18.7)	214 (25.6)		
Sex, Male	503 (81.3)	108 (76.6)	49 (65.3)	660 (79.0)	0.004	0.208
Ethnicity, Arab/Other	106 (17.1)	19 (13.5)	21 (28.0)	146 (17.5)	0.025	0.291
Cardiac diseases						
Cardiomegaly	78 (12.6)	25 (17.7)	8 (10.7)	111 (13.3)	0.211	0.108
Supraventricular arrhythmias	111 (17.9)	29 (20.6)	17 (22.7)	157 (18.8)	0.515	0.466
Congestive heart failure	161 (26.0)	46 (32.6)	19 (25.3)	226 (27.1)	0.263	0.111
Pulmonary heart disease	52 (8.4)	16 (11.3)	6 (8.0)	74 (8.9)	0.519	0.269
Chronic ischemic heart disease	558 (90.1)	129 (91.5)	68 (90.7)	755 (90.4)	0.885	0.625
Previous myocardial infarction	138 (22.3)	31 (22.0)	16 (21.3)	185 (22.2)	0.981	0.937
Previous percutaneous coronary intervention	143 (23.1)	31 (22.0)	12 (16.0)	186 (22.3)	0.376	0.776
Previous coronary artery bypass graft	57 (9.2)	12 (8.5)	8 (10.7)	77 (9.2)	0.873	0.795
Atrioventricular block	29 (4.7)	5 (3.5)	2 (2.7)	36 (4.3)	0.637	0.555
Cardiovascular risk factors						
Diabetes mellitus	287 (46.4)	80 (56.7)	41 (54.7)	408 (48.9)	0.048	0.026
Dyslipidemia	521 (84.2)	117 (83.0)	64 (85.3)	702 (84.1)	0.896	0.728
Hypertension	418 (67.5)	98 (69.5)	48 (64.0)	564 (67.5)	0.713	0.650
Obesity	146 (23.6)	44 (31.2)	25 (33.3)	215 (25.7)	0.051	0.059
Smoking	268 (43.3)	61 (43.3)	29 (38.7)	358 (42.9)	0.742	0.994
Peripheral vascular disease	97 (15.7)	33 (23.4)	10 (13.3)	140 (16.8)	0.060	0.028

Family history of ischemic heart disease	61 (9.9)	7 (5.0)	9 (12.0)	77 (9.2)	0.133	0.066
**Other disorders**						
Chronic obstructive pulmonary disease	62 (10.0)	17 (12.1)	8 (10.7)	87 (10.4)	0.772	0.474
Neurological disorders	109 (17.6)	26 (18.4)	10 (13.3)	145 (17.4)	0.610	0.816
Malignancy	34 (5.5)	6 (4.3)	2 (2.7)	42 (5.0)	0.514	0.553
Anemia	386 (62.4)	102 (72.3)	46 (61.3)	534 (64.0)	0.074	0.026
Gastrointestinal bleeding	25 (4.0)	11 (7.8)	2 (2.7)	38 (4.6)	0.110	0.058
Schizophrenia/Psychosis	20 (3.2)	3 (2.1)	0	23 (2.8)	0.240	0.784
Alcohol/drug addiction	17 (2.7)	5 (3.5)	1 (1.3)	23 (2.8)	0.639	0.581
History of malignancy	32 (5.2)	8 (5.7)	3 (4.0)	43 (5.1)	0.868	0.809
**Administrative characteristics of the hospitalization**						
Admitted/transposed to ICCU	445 (71.9)	97 (68.8)	47 (62.7)	589 (70.5)	0.225	0.463
Length of hospital stay, days, Mean (SD)	16.54 (11.52)	23.44(16.75)	13.86 (8.51)	17.47 (2.64)	<0.001	<0.001
≥7	511 (82.6)	136 (96.5)	63 (84.0)	710 (85.0)	<0.001	<0.001
**Clinical characteristics of the hospitalization**						
Type of AMI, STEMI	296 (47.8)	59 (41.8)	33 (44)	388 (46.5)	0.379	0.199
**Results of echocardiography**						
Echocardiography performance	500 (80.8)	119 (84.4)	55 (73.3)	674 (80.7)	0.145	0.318
Severe left ventricular dysfunction	91 (18.2)	27 (22.7)	11 (20.0)	129 (19.1)	0.527	0.263
Left ventricular hypertrophy	23 (4.6)	10 (8.4)	2 (3.6)	35 (5.2)	0.210	0.097
Mitral regurgitation	42 (8.4)	12 (10.1)	0	54 (8.0)	0.061	0.558
Tricuspid regurgitation	23 (4.6)	5 (4.2)	1 (1.8)	29 (4.3)	0.627	0.851
Pulmonary hypertension	45 (9.0)	18 (15.1)	6 (10.9)	69 (10.2)	0.138	0.047
**Results of angiography**						
Angiography performance	455 (73.5)	104 (73.8)	49 (65.3)	608 (72.8)	0.311	0.951
Measure of coronary artery disease						
No/non-significant	6 (1.3)	3 (2.9)	0	9 (1.5)	0.681	0.455
One vessel	58 (12.7)	9 (8.7)	5 (10.2)	72 (11.8)		

Two vessels	86 (18.9)	19 (18.3)	11 (22.4)	116 (19.1)		
Three vessels/Left main artery	305 (67)	73 (70.2)	33 (67.3)	411 (67.6)		
Type of treatment						
Noninvasive	94 (15.2)	14 (9.9)	13 (17.3)	121 (14.5)		
Percutaneous coronary intervention	220 (35.5)	35 (24.8)	27 (36.0)	282 (33.8)	0.013	0.003
Coronary artery bypass graft	305 (49.3)	92 (65.2)	35 (46.7)	432 (51.7)		

(b)

Recovery group *	(1)<br>Early recovery	(2)<br>No early recovery	(3)<br>Missing values of serum Creatinine	Total	p<br>(Overall)	p<br>(1 vs. 2)
n	98	34	102	234		
Demographics						
Age, Years, Mean (SD)	67.94 (11.99)	68.59 (11.04)	67.91 (13.05)	68.02 (12.29)	0.959	0.792
<65	35 (35.7)	12 (35.3)	39 (38.2)	86 (36.8)		
65-75	35 (35.7)	12 (35.3)	30 (29.4)	77 (32.9)	0.905	0.996
≥75	28 (28.6)	10 (29.4)	33 (32.4)	71 (30.3)		
Sex, Male	59 (60.2)	21 (61.8)	56 (54.9)	136 (58.1)	0.672	0.873
Ethnicity, Arab/Other	14 (14.3)	7 (20.6)	13 (12.7)	34 (14.5)	0.530	0.387
Cardiac diseases						
Cardiomegaly	13 (13.3)	4 (11.8)	20 (19.6)	37 (15.8)	0.368	0.822
Supraventricular arrhythmias	34 (34.7)	6 (17.6)	23 (22.5)	63 (26.9)	0.064	0.062
Congestive heart failure	28 (28.6)	13 (38.2)	36 (35.3)	77 (32.9)	0.464	0.294
Pulmonary heart disease	17 (17.3)	2 (5.9)	15 (14.7)	34 (14.5)	0.262	0.101
Chronic ischemic heart disease	77 (78.6)	30 (88.2)	85 (83.3)	192 (82.1)	0.406	0.215
Previous myocardial infarction	23 (23.5)	14 (41.2)	22 (21.6)	59 (25.2)	0.065	0.048
Previous percutaneous coronary intervention	18 (18.4)	7 (20.6)	18 (17.6)	43 (18.4)	0.929	0.776
Previous coronary artery bypass graft	11 (11.2)	7 (20.6)	5 (4.9)	23 (9.8)	0.024	0.17
Atrioventricular block	4 (4.1)	1 (2.9)	7 (6.9)	12 (5.1)	0.553	0.764
Cardiovascular risk factors						
Diabetes mellitus	50 (51.0)	25 (73.5)	52 (51)	127 (54.3)	0.051	0.022
Dyslipidemia	76 (77.6)	28 (82.4)	85 (83.3)	189 (80.8)	0.565	0.555
Hypertension	65 (66.3)	24 (70.6)	70 (68.6)	159 (67.9)	0.883	0.648
Obesity	25 (25.5)	7 (20.6)	26 (25.5)	58 (24.8)	0.829	0.564
Smoking	39 (39.8)	14 (41.2)	37 (36.3)	90 (38.5)	0.825	0.887
Peripheral vascular disease	22 (22.4)	10 (29.4)	19 (18.6)	51 (21.8)	0.410	0.414
Family history of ischemic heart disease	8 (8.2)	1 (2.9)	5 (4.9)	14 (6.0)	0.449	0.298

**Other disorders**						
Chronic obstructive pulmonary disease	15 (15.3)	5 (14.7)	17 (16.7)	37 (15.8)	0.948	0.933
Neurological disorders	27 (27.6)	10 (29.4)	26 (25.5)	63 (26.9)	0.890	0.835
Malignancy	5 (5.1)	1 (2.9)	5 (4.9)	11 (4.7)	0.870	0.602
Anemia	66 (67.3)	30 (88.2)	64 (62.7)	160 (68.4)	0.021	0.018
Gastrointestinal bleeding	6 (6.1)	2 (5.9)	5 (4.9)	13 (5.6)	0.928	1.000
Schizophrenia/Psychosis	4 (4.1)	0	1	5 (2.1)	0.205	0.572
Alcohol/drug addiction	1	0	1	2 (0.9)	0.842	1.000
History of malignancy	4 (4.1)	0	5 (4.9)	9 (3.8)	0.431	0.232
**Administrative characteristics of the hospitalization**						
Admitted/transposed to ICCU	72 (73.5)	24 (70.6)	71 (69.6)	167 (71.4)	0.828	0.745
Length of hospital stay, days, Mean (SD)	30.58 (25.00)	40.73 (35.07)	20.86 (16.29)	27.82 (24.46)	<0.001	0.031
≥7	94 (95.9)	34 (100.0)	92 (90.2)	220 (94.0)	0.066	0.572
**Clinical characteristics of the hospitalization**						
Type of AMI, STEMI	40 (40.8)	13 (38.2)	41 (40.2)	94 (40.2)	0.966	0.791
**Results of echocardiography**						
Echocardiography performance	78 (79.6)	27 (79.4)	80 (78.4)	185 (79.1)	0.978	0.982
Severe left ventricular dysfunction	22 (28.2)	10 (37)	16 (20)	48 (25.9)	0.182	0.39
Left ventricular hypertrophy	6 (7.7)	1 (3.7)	5 (6.3)	12 (6.5)	0.764	0.674
Mitral regurgitation	4 (5.1)	1 (3.7)	6 (7.5)	11 (5.9)	0.711	1.000
Tricuspid regurgitation	6 (7.7)	0	5 (6.3)	11 (5.9)	0.342	0.335
Pulmonary hypertension	10 (12.8)	2 (7.4)	7 (8.8)	19 (10.3)	0.609	0.727
**Results of angiography**						
Angiography performance	69 (70.4)	20 (58.8)	73 (71.6)	162 (69.2)	0.358	0.214
Measure of coronary artery disease						
No/non-significant	2 (2.9)	1 (5.0)	3 (4.1)	6 (3.7)		
One vessel	7 (10.1)	4 (20.0)	15 (20.5)	26 (16.0)	0.389	0.612
Two vessels	19 (27.5)	4 (20.0)	10 (13.7)	33 (20.4)		

Three vessels/Left main artery	41 (59.4)	11 (55.0)	45 (61.6)	97 (59.9)		
**Type of treatment**						
Noninvasive	23 (23.5)	10 (29.4)	19 (18.6)	52 (22.2)		
Percutaneous coronary intervention	34 (34.7)	9 (26.5)	36 (35.3)	79 (33.8)	0.683	0.634
Coronary artery bypass graft	41 (41.8)	15 (44.1)	47 (46.1)	103 (44.0)		

Data are presented as numbers (percentage), unless specified otherwise. *Recovery groups were defined as: 'Rapid recovery' - patients with creatinine ratio <1.5 at 12-36 hours and/or <1.45 at 36-60 hours after AKI diagnosis; 'No rapid recovery' - patients with creatinine ratio ≥1.5 at 12-36 hours and ≥1.45 at 36-60 hours after AKI diagnosis; 'Early recovery' - patients with creatinine ratio <2.50 at 84-108 hours after AKI diagnosis; 'No early recovery' - patients with creatinine ratio ≥2.50 at 84-108 hours after AKI diagnosis. AMI – Acute myocardial infarction, ICCU – Intensive cardiac care unit, SD – standard deviation, STEMI - ST Elevation myocardial infarction.
